# Virtual reality and haptic simulation in modern microsurgical endodontics: Case report and proof of concept

**DOI:** 10.1111/iej.14239

**Published:** 2025-04-19

**Authors:** Damiano Pasqualini, Giorgia Carpegna, Mario Alovisi, Elio Berutti, Sami Chogle

**Affiliations:** ^1^ Department of Surgical Sciences, Dental School Endodontics Unit, University of Turin Turin Italy; ^2^ Department of Endodontics Boston University Henry M. Goldman School of Dental Medicine Boston Massachusetts USA

**Keywords:** endodontics, haptic, microsurgical endodontics, virtual reality simulation, virtual training

## Abstract

**Aim:**

To highlight the development and application of a novel virtual reality (VR) haptic simulation program in endodontic microsurgery (EMS) to prepare for a clinical case performed by a resident student.

**Summary:**

Modern EMS requires adequate training and a learning curve for mastering surgical techniques and refining individual skills. VR haptic simulation is gaining attention in dentistry. Pre‐clinical virtual training may enhance skills and minimize the risk of unintentional iatrogenic damage during surgery. VR simulation offers an innovative approach, allowing for the saving, reviewing and repetition of exercises without constraints.

In this case report, a female patient presented with persistent apical periodontitis related to an endodontically treated maxillary right second premolar. Utilizing a validated digital workflow, cone beam‐computed tomography images were converted into STL file format and optimized for VR simulation. The Virteasy Editor interface was used to generate graphics and touchable haptic solids representing different tissues (enamel, dentine, root canal filling and bone). Following the creation of the virtual patient and the simulation of the endodontic lesion, a postgraduate student in endodontics executed 10 sessions of ostectomy, root‐end resection and ultrasonic retro preparation in the evaluation stage until reaching ideal standards of practice in the virtual scenario. The student then proceeded to perform supervised root‐end surgery on the actual patient. A 1‐year postoperative CBCT evaluated the healing outcomes.

## INTRODUCTION

Endodontic microsurgery (EMS) is a surgical option for the preservation of natural dentition in patients with symptoms and clinical and radiological signs of lesions of endodontic origin. Most periapical lesions should respond positively to non‐surgical endodontic retreatment, which is considered the option of choice in case of inadequate primary root canal treatment, missing canals or loss of coronal seal (Setzer & Kratchman, 2022). Otherwise, EMS is indicated as an alternative treatment option when non‐surgical endodontic retreatment fails or is deemed to be unfeasible (Stueland et al., 2023). Therefore, severe alterations of the canal anatomy, posts, perforations and resorptions could be considered factors suggesting the necessity of a surgical approach (Setzer & Kratchman, 2022). Modern EMS integrates the use of an operating microscope, a minimally invasive osteotomy, zero‐degree bevel root‐end resection, ultrasonic‐assisted root‐end preparation and biocompatible root‐end filling materials (Kim & Kratchman, 2006). Even so, a comprehensive work‐up to identify both aetiology and surgical feasibility still necessitates high operator skill to achieve predictable outcomes (Torabinejad & White, 2016). The EMS success rates vary from 78% to 92% (Song et al., 2012; von Arx et al., 2012) influenced by tooth‐related prognostic variables including lesion size and periodontal status (Song et al., 2013).

Among all outcome predictors, the presence of a ‘major procedural error’ during surgery in ostectomy for root apex identification and root end management is the most significant (Azim et al., 2021). Incorrect root end management and retro filling are considered the major reasons for reintervention after failed apical surgery (Song et al., 2011).

Data from 1045 surveys of active AAE members indicate that access and visualization, followed by root‐end management and filling, are considered the most difficult stages of surgery (Creasy et al., 2009). Of those surveyed, 33.3% felt that their endodontic residency training in surgery was inadequate, and 47.9% went on to acquire additional training in surgery post residency. Most respondents would positively consider additional advanced learning through new technologies.

The conventional approach relies entirely on a freehand (FH) technique, preoperative cone beam computed tomography (CBCT) and operator's expertise (Ee et al., 2014). This approach is operator‐dependent and may be subject to several difficulties and risks of complications.

Cone beam computed tomography technology has significantly expanded the treatment options in EMS with the introduction of guided surgical techniques (Giacomino et al., 2018; Hawkins et al., 2020) and dynamic navigation systems (DNS) (Mekhdieva et al., 2023; Tang & Jiang, 2023) into clinical practice.

This new approach may reduce operation time with safe and accurate real‐time tracking and adjustability during surgical steps (Chong et al., 2019). Although DNS and guided EMS offer new possibilities, these techniques require further operator training and equipment. As a result, the FH approach remains the gold standard and the most utilized. Pre‐operative planning and training are fundamental to understanding the complexity of the endodontic and surgical site anatomy and may reduce the risk of iatrogenic errors. In fact, apical and coronal deviations, missing or perforating the apex, can lead to suboptimal results or complications (Rubinstein & Kim, 1999).

Among preclinical training options, physical 3D‐printed jaw models (Anderson et al., 2018; Bahcall, 2014) or cadaver lab training are probably closer to clinical reality. However, they are irreversible and destructive procedures affected by high costs for repetitive training.

Computer simulations and mixed reality (augmented and virtual reality) are demonstrating a huge potential and stimulating increased attention in dental education and clinical practice (Joda et al., 2019), as they may provide, after the significant initial investment costs for the simulator and software, a new and non‐destructive approach, allowing the operator to practice and repeat procedures without limitations and at no incremental cost. As a determinant of the learning outcome, physical interactivity with the virtual scenario through haptic technology provides tactile force feedback to the user and a more realistic experience (Dixon et al., 2021). There are different types of haptic feedback: cutaneous, which refers to pressure shear and vibrations applied to the skin (e.g. Braille cells) and kinesthetic, which involves forces and motion sensed by the muscle's tendons and joints (e.g. virtual simulators). Both cutaneous and kinesthetic may be active where the user is in control of his action and consciously applies forces or motion to the surface or they may be passive which allows a device to impose the information or guide the user's actions (Rodríguez et al., 2019). There are two popular control methods widely used in haptic controller design. The first is impedance control, where the operator's motion input is measured, and the reaction force is fed back to the operator. The alternative method is admittance control, where forces exerted by the operator are measured, and positions are fed back to the operator. Both impedance and admittance control are also basic methods for interacting with a virtual environment (Wen et al., 2005).

Virtual reality (VR) haptics in dental training offer an effective and innovative approach to simulating the technical and emotional aspects of the clinical experience. It allows for the reproduction of a real clinical case through digital planning, virtual scenarios and haptic feedback, enabling the saving and reviewing of the trainer's progress, as well as the assessment of performance and outcomes at any time (Wang et al., 2015).

Haptic virtual reality simulation has been proposed in endodontics for access cavity preparation training (Suebnukarn et al., 2011) to improve operative skills in endodontic treatment (Suebnukarn et al., 2010) and local anaesthesia (Correa et al., 2017). Augmented reality with superimposition of pre‐generated virtual 3D content on real surgical sites has been applied in endodontic microsurgery as an alternative option to guided osteotomy and apex location and identification (Chen et al., 2023). VR haptic simulation has been introduced for oral surgery procedures, including traditional apical surgery with simulation of bone removal using a rotating handpiece and of infected tissue around the root tip of the tooth with the surgical spoon (Buchbender et al., 2021). To date, there are no simulators implementing VR haptic scenarios based on the principles of modern endodontic microsurgery.

The aim of this case report is to illustrate the first creation of a novel VR haptic simulation in modern endodontic microsurgery and its application to adequately train a low expertise level operator, a resident student in endodontics, before the execution of supervised EMS intervention in a real clinical case on a patient.

## CASE REPORT

**FIGURE 1:**
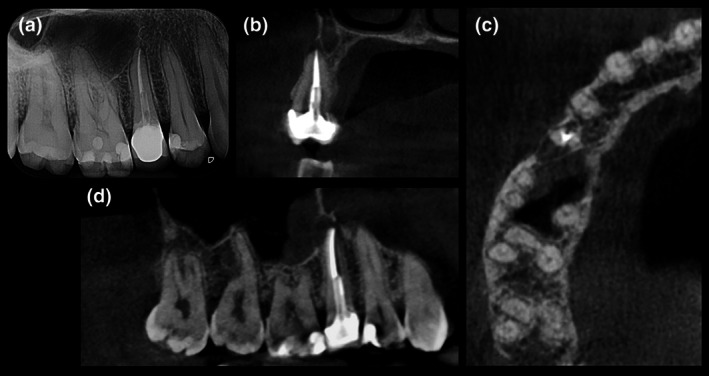
(a) Pre‐operative intraoral radiograph; (b–d) Pre‐operative CBCT, coronal, horizontal axial, sagittal view showing apical radiolucency of tooth 1.5 and inflammatory reaction of the maxillary sinus.

This case report has been written according to Preferred Reporting Items for Case reports in Endodontics (PRICE) 2020 guidelines. A 34‐year‐old white‐Caucasian woman presented at the Endodontics Unit of the University of Turin Dental School reporting swelling and spontaneous pain in the right upper maxilla. The patient's medical and dental histories were collected. Pre‐operative digital periapical radiograph (Figure 1) and cone beam‐computed tomography (Planmeca Promax 3D, Planmeca, Helsinki, Finland; FOV 8 × 8 cm, voxel size ≤200 μm, 200° scan) (Figure 1b–d) confirmed signs of apical abscess associated with a previously root canal treated tooth 1.5, restored with a fibre post, composite resin core and a metal‐ceramic crown. The tooth was adjacent to the anterior wall of the maxillary sinus with evidence of an inflammatory reaction. The buccal cortical bone was mostly intact with a thin plate at the root apex. Periodontal probing indicated healthy periodontal status with no detectable vertical root fractures. The case was presented to the patient including treatment options and their risks and benefits. Informed written consent was obtained, and EMS was assigned to a postgraduate endodontic resident.

The resident's training in endodontic microsurgery was through 3D printed models, a cadaver‐based lab training, and as an observer of live surgeries performed by an expert operator. As an additional learning experience, a novel virtual reality (VR) and haptic simulation program was introduced to the post‐graduate student utilizing a previously tested VR digital workflow (Carpegna et al., 2023) created by the authors.

This workflow includes

1. Generation of digital data;
2. Creation of the VR dental simulation;
3. Assessment criteria and feedback statements.

Cone beam computed tomography volumes, along with clinical information of the tooth and jaw, were exported in an STL format to prepare and optimize shape and shade for the VR dental simulation. The STL data sets generated from the scans were processed using Geomagic Qualify 2015 (3D Systems, Rock Hill, SC, USA), Materialize Mimics 3‐Matic (Materialize, Leuven, Belgium) and Blender 3.1.2 (Blender Foundation, Amsterdam, Netherlands). To generate a haptic object, a voxelization algorithm transformed surfaces into voxel‐made 3D objects with a voxel size of 0.10 mm, followed by a smoothing algorithm to transform cubic voxels into smooth shapes for more realistic feedback. Graphics and touchable haptic solids were created through the Virteasy Editor interface (HRV, Laval, France), which allows the transformation of 3D surfaces (STL, PLY formats) into graphical and volumetric haptic solids based on their density (enamel, dentine, pulp, bone). The elements were then exported into a graphical scene within a virtual patient (avatar), which can be interacted with via a haptic device for treatment purposes. Assessment criteria were determined, and feedback statements were created using a questionnaire with fixed answer options (Data S1—questionnaire), enabling the software to provide qualitative feedback on osteotomy design and extension, bevel of root end resection and retrograde preparation.

**FIGURE 2:**
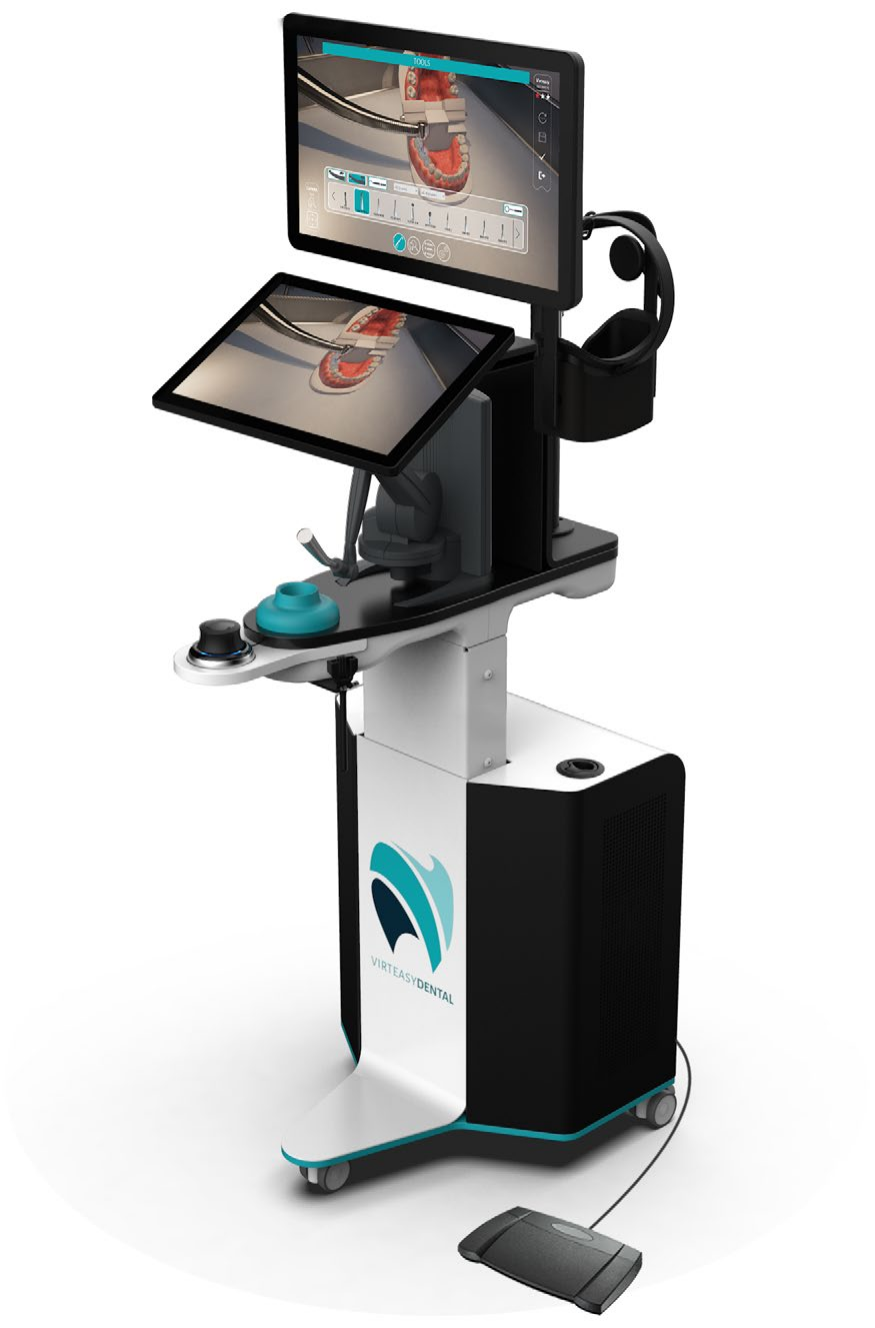
Virteasy Dental PRO/V2 is a high‐fidelity dental training simulator with the capability to use VR headsets for a fully immersive environment and haptic devices to provide force feedback and realistic sensations of the simulated treatments. The hardware is provided with a touch screen for navigation and an immersive screen for the user. Beneath the lower screen is the haptic device, which provides force feedback up to 7.9 N forces and with 6 degrees of freedom (DOF) positional sensing and 3 DOF force feedback, which allows the manipulation and feeling of real dental tools. Equally, a Polhemus Patriot tracking device is available to the user to mimic the use of a dental mirror and to provide indirect vision. Optionally, the user can use a virtual reality headset, the META Quest 3, for a fully immersive environment that offers a 360° simulation environment without distractions.

The VR dental learning environment room was set up using specific VR units (V2 HRV, Laval, France) (Figure 2) with dedicated haptic hardware and a software interface (Virteasy, HRV, Laval France), in the Virtual Training Dental Centre of the University of Turin Dental School (Video S2).

**FIGURE 3:**
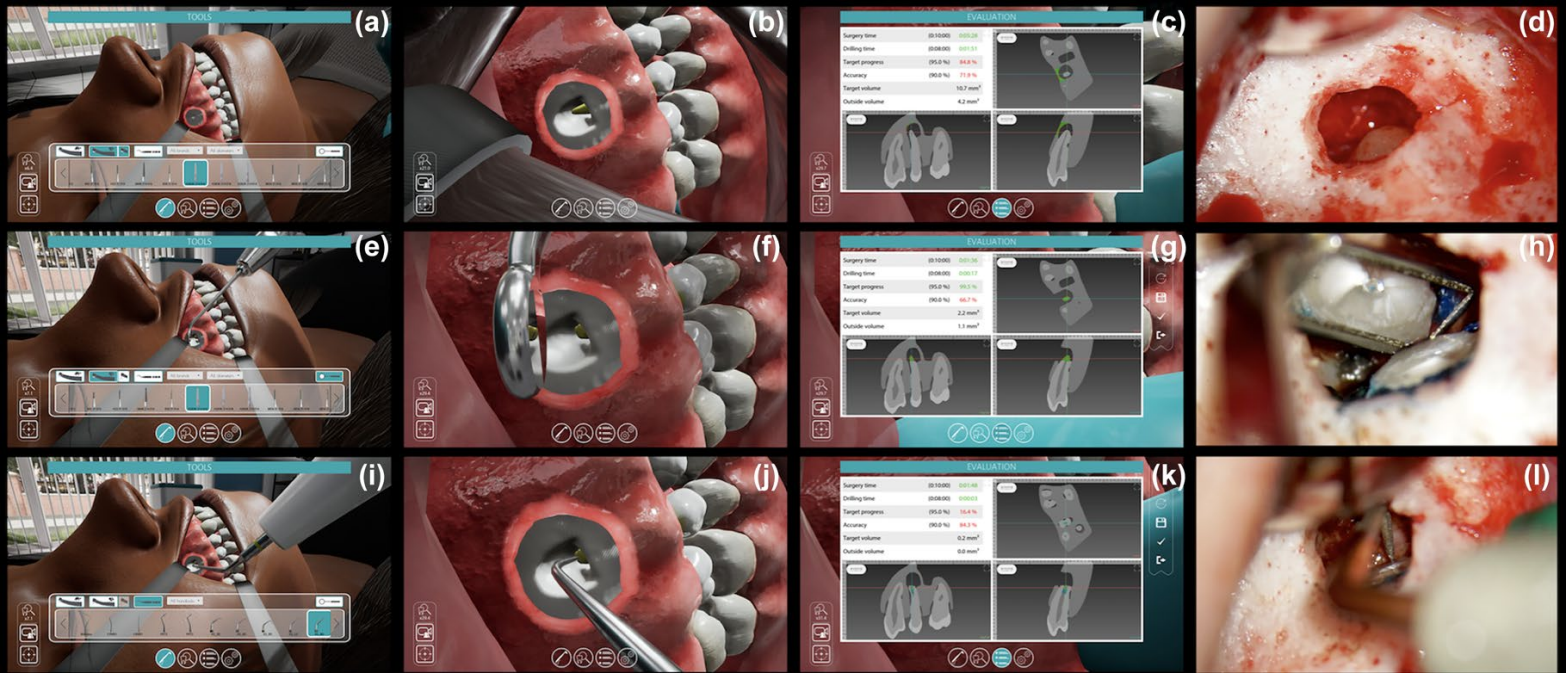
(a, b) Virtual scenario before and after the ostectomy; (c) real‐time 3D planes evaluation; (d) clinical view of ostectomy and root apex localization; (e–f) virtual scenario before and after the apicoectomy; (g) real‐time 3D planes evaluation; (h) clinical view of root end resection; (i, j) virtual scenario before and after the ultrasonic retro cavity preparation; (k) real‐time 3D planes evaluation; (l) clinical view of ultrasonic retro cavity preparation.

As requested by evaluation criteria implemented in the software interface, the student was asked to execute in the VR haptic scenario: the access bone window (Figure 3a,b) of 5 × 5 mm with localization of the root tip in the centre; the complete root end resection of 3 mm from the apex (1) with a bevel angle of 10° (Figure 3e,f), considered the optimal average target in the range of 0°–20° (von Arx et al., 2016); the ultrasonic retro preparation (Figure 3i,j) of 3 mm depth (1) aligned with the root canal and long axis of the root in buccal‐lingual and mesial‐distal dimensions. In the simulation, a high‐speed dental handpiece, a multi‐blade bur and an ultrasonic handpiece with specific retro tips were selected from the tool menu. A virtual magnification tool allowed the user to zoom in (and back out) at increments of 1× (default), 2×, 4×, etc., up to 20×. A dental probe and a micromirror for indirect vision were also available. The student was allowed to practice a few times without evaluation to familiarize himself with the VR scenario. When the student reached a minimum level of self‐confidence with the equipment and environment, he started the evaluation stage for a total of 10 attempts, until meeting the requirements assessed by the software. During the evaluated simulation, the student was allowed for the first five attempts to visualize the guidance of the ideal target area and volume (evidenced in blue colour in the VR scenario), while the guidance was deactivated in the following five attempts. The operator could also check in real time on multiple planes the target progress and accuracy (Figure 3c,g,k). The resident was required to achieve 95% (as the highest and most stringent limit of the assessment criteria, with a tolerance of 10%) in target progress (i.e. the percentage of tissue removed compared to the predefined volume; it is directly proportional to the amount of tissue removed) and 90% (as the highest and most stringent limit of the assessment criteria, with a tolerance of 10%) in accuracy (i.e. the percentage of tissue removed outside the target volume; it is inversely proportional to the amount of tissue removed). In the simulation, the accidental removal of bone from the thin lateral wall of the maxillary sinus adjacent to the endodontic lesion was considered by the software as an error, negatively impacting the parameter ‘accuracy’.

The outcome and feedback of each session were recorded to help the student better appreciate the improvements in the learning curve. Moreover, the exercises were reviewed by a clinical academic in endodontics and discussed with the student at the end of each session. When the student was able to reach the requested performance criteria, the supervised microsurgical clinical procedure was approved.

After local anaesthesia (articaine with adrenaline 1:100 000) and haemostasis (lidocaine with adrenaline 1:50 000), a mucoperiosteal, triangular, papilla‐based flap was performed and elevated under a surgical microscope (Zeiss OPMI Pro Ergo, Carl Zeiss Meditec, Inc., Oberkochen, Germany) (Velvart, 2002; Velvart et al., 2005). The osteotomy was achieved with a high‐speed handpiece under water spray with a surgical Lindemann bur to visualize the root, lesion and bony crypt (Figure 3d). Subsequently, the curettage of the lesion and the completion with the same bur of the root‐end resection with a shallow bevel angle of <20° were performed (Figure 3h). Ferric sulfate 14% with a sterile cotton pellet was placed in the bony crypt for further haemostasis. After evidence with methylene blue of the root end area, apical retro‐preparation was carried out with an ultrasonic tip (Endo Success Satelec) (Figure 3l) and filled with bioceramic material (RRM Fast Set Putty Endosequence, Brasseler, USA). Finally, the flap was passively repositioned by primary intention, gently compressed with sterile gauze, and sutured. The patient received post‐operative instructions and analgesic prescriptions.

Post‐operative digital periapical radiograph and cone beam‐computed tomography with same settings of baseline were collected at 1 year for healing assessment and comparison.

**FIGURE 4:**
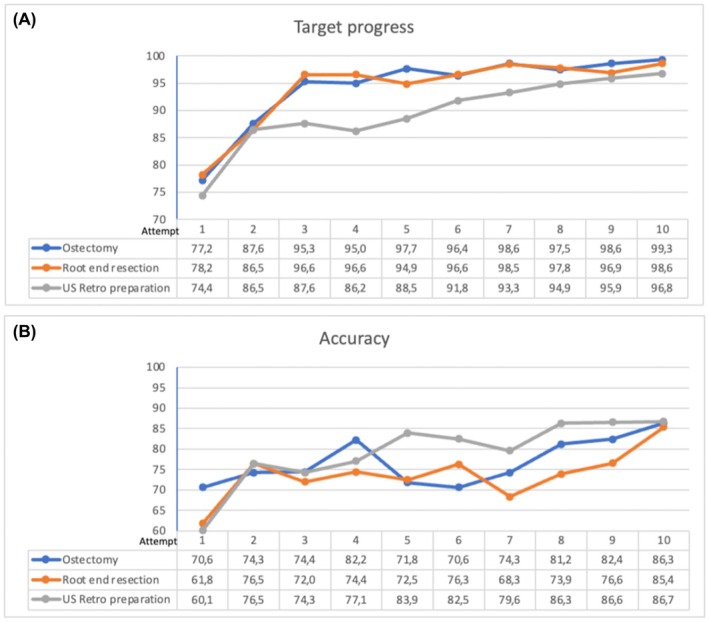
(a) Comparison of the operator's values of target progress from the first and last attempt without target. (b) Comparison of the operator's values of accuracy from the first and last attempts without target.

Figure 4 reports the curve of performance improvements of the operator in target progress and accuracy evaluation parameters from first attempt until reaching requirements for the surgical stage at attempt no. 10.

**FIGURE 5:**
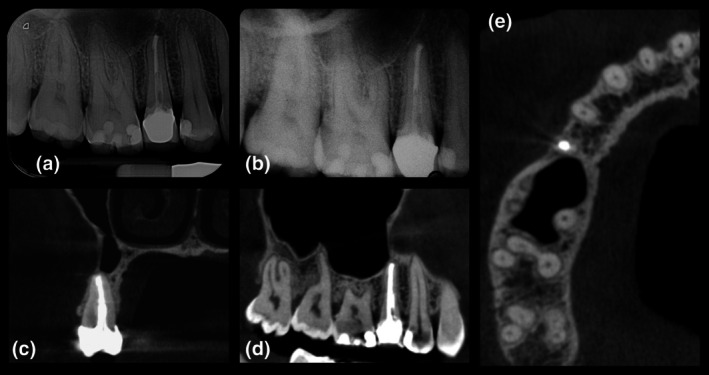
(a) Immediate post‐operative baseline; (b) one‐year follow‐up intraoral radiograph; (c–e) CBCT at 1 year, coronal, sagittal, horizontal axial view showing complete healing of the lesion and maxillary sinus.

At 1 year follow‐up the patient was asymptomatic and periapical radiograph and cone beam‐computed tomography showed complete healing of the periapical lesion with reformation of the buccal cortical plate and healing of the maxillary sinus (Figure 5d). The management of the root end was judged as satisfactory both in bevel angle and retro cavity preparation and filling. This case report suggests the possibility of providing reliable and clinically relevant qualitative feedback with an appropriate VR scenario and haptic simulator for a trainer program in modern microsurgical endodontics.

## DISCUSSION

This case report highlights the potential benefits of a novel haptic virtual reality simulation in the clinical training in modern endodontic microsurgery. A relatively inexperienced operator could improve their level of confidence and technical skills in a case‐specific virtual environment before executing the EMS intra‐orally with reproducible results. The possibility of viewing 3D learning virtual content multiple times and the experience of tactile feedback from the haptic device may provide the opportunity for operators to increase their level of competency and proficiency and develop manual skills. The feedback from the operator after the VR experience and its immediate translation into clinical practice was highly positive, as suggested by the improvement of confidence and skills through the virtual simulation (Figure 3a,b).

The application of VR in dental education has significantly evolved, and there is increasing evidence supporting the advantages of different virtual setups. A diverse array of devices and technologies is now feasible: VR simulations with or without immersive environments, haptic simulators with or without force feedback, AR devices, real‐time digital mapping and evaluation, virtual mobile platforms, video games and various other virtual packages. The varied and detailed features of these individualized approaches highlight the absence of established educational standards for dental simulators or related exercises (Moussa et al., 2022). VR surgical training may improve operator performance and reduce the rate of surgical complications compared to outcomes in animal (Suebnukarn et al., 2012) and human cadaver models (von Sternberg et al., 2007). Moreover, VR haptic simulation contributes to the progressive entrustment of trainees by providing a structured environment in which they can develop confidence and technical skills before performing actual clinical procedures. Furthermore, practicing in a virtual environment eliminates the risk of harm to patients, allowing users to make mistakes and learn from them without real‐world consequences.

The Virteasy Dental (HRV, Laval, France) teaching and learning facility utilized in this case report is a VR haptic interactive system designed for education and practical training. The computerized virtual environment combines an avatar patient model with a library of dental procedures, permitting the performance of different tasks and exercises. The simulator also has the option to activate a virtual magnification tool that allows the user to zoom in (and back out) at increments of 1× (default), 2×, 4×, etc., up to 20×. It is managed by modifying the field of view and distance of the virtual camera. It is not a physically accurate depiction of a microscope (especially because depth of field is not managed), however, this gives the approximation of using loupes or a microscope in real life.

The Virteasy simulator can be utilized in a VR immersive setting with a headset, or in a semi‐immersive setting by watching at a regular monitor without a VR headset. The user can properly position around the virtual patient and also adjust the position of the virtual patient (height, inclination of the chair, etc.) through a joystick. The virtual magnification tool is available in both semi‐immersive and ‘VR’ mode. In ‘VR’ mode, this feature works slightly differently in order to avoid motion sickness that occurs when the feature is applied for the whole display. To counteract this, Virteasy Dental uses a secondary tool, very similar to tablet devices, to provide a static magnification. The result is very similar to a microscope where the user looks directly into the secondary device to see a magnified version of the simulation. This helps reduce the effects of motion sickness and provides a close approximation to traditional dental magnification devices.

Moreover, the program allows a computerized assessment system that is reliable and objective to help standardize feedback and evaluation.

The VR haptic simulation presented in this case report allowed for managing and training the optimal surgical approach and spatial visualization of the surgical field, improving manual skills and effectively learning surgical principles. The pre‐operative planning and VR experience might also have improved the confidence of the operator with a more effective prevention of iatrogenic errors, such as perforation of the adjacent maxillary sinus, which may occur in 10% to 50% of cases (Freedman & Horowitz, 1999), as well as incorrect root‐end preparation (Setzer et al., 2021; Song et al., 2011).

Appropriate targeting of the root apex through a precise localization and execution of the bone window, a 3 mm root apex resection and a shallow bevel angle (0°–20°), the ultrasonic retro‐cavity preparation at least 3 mm deep and aligned with the root canal axis, are considered the gold standard for root end management (Kim & Kratchman, 2006). However, these stages are perceived as technically demanding, and even expert operators may not always achieve the ideal outcome (von Arx et al., 2012). The settings and computerized assessment criteria of the present VR haptic simulation were configured according to the mentioned best standards of practice in modern surgical microendodontics (Kim & Kratchman, 2006). Target progress and accuracy were the two main evaluation parameters of students' performance and improvement during the training program. They are digital metrics used by the simulator to keep track of the progress of the user in the exercise. “Target progress” corresponds to how much of the defined target was removed. A “target” is a volume defined in the haptic object that corresponds to the ideal preparation to be performed. “Accuracy” is a percentage to show the precision of the student. It is computed by the volume (in mm^3^) of removed/drilled target tissue divided by the total removed/drilled volume. If the student has removed only target material, then the accuracy is 100%. If the student has only removed some volume that is not defined in the target volume, then the accuracy is 0%.

The student demonstrated a significant improvement in both parameters from baseline performance to final results, evidencing a favourable and effective learning curve as shown in Figure 4.

In this case report, the surgical procedure performed after haptic VR simulation showed a successful outcome at 1‐year follow‐up (Figure 5d).

Virtual reality simulators present an innovative tool for medical and dental teaching; however, their impact in clinical learning and improvement of clinical competency is still considered controversial. In minimally invasive general surgery, VR simulations have been successfully utilized, demonstrating a beneficial impact of warm‐up on the laparoscopic performance of surgeons before entering the operating room (Calatayud et al., 2010). Furthermore, haptic feedback systems can improve the performance and accuracy of expert surgeons in robot‐assisted surgery and reduce the learning time needed by new surgeons (El Rassi & Rassi, 2020).

The impact on effective learning and the advantages of virtual simulation compared to traditional training on 3D printed models in endodontics are still controversial and considered complementary to traditional methods (Duan et al., 2024). However, many aspects are gaining increasing interest among the dental and endodontic educational community. VR and haptic simulations provide an interactive and immersive experience, allowing dental students and professionals to practice procedures in a realistic virtual environment; this can enhance learning and skill acquisition by simulating real‐life scenarios. While the initial setup for VR and haptic systems can be expensive, they can be more cost‐effective in the long run. Once the system is in place, it can be used repeatedly without the need for physical materials, unlike 3D printed models which require materials for each new model. VR systems can be easily scaled and distributed, allowing multiple users to access the same training modules simultaneously, regardless of their location. This is particularly beneficial for remote learning and training. Haptic simulations provide real‐time tactile feedback, which can help users develop a better sense of touch and pressure, crucial for dental procedures. This immediate feedback can accelerate the learning process and improve technique. Furthermore, virtual environments can be easily customized to simulate a wide range of dental conditions and procedures, offering a broader scope of training scenarios than static 3D printed models. VR systems can also track user performance and provide detailed analytics, helping educators to assess the progress and identify the areas for improvement. This data‐driven approach can enhance the effectiveness of training programs. The acquisition of basic dental motor skills is best optimized through the combination of instructor and visual display VR‐driven feedback (Al‐Saud et al., 2017). VR is also effective in reducing anxiety linked to real patient management during treatment planning; it provides an interactive learning experience, enhances self‐assessed competence and boosts confidence in dealing with real patients (Clark et al., 2012). Simulators, offering flexibility in terms of time, enable the repetition of procedures until acceptable skill levels are attained without putting real patients at risk or necessitating prolonged direct contact (Mardani et al., 2020).

The ability to view 3D teaching content several times makes it easier to memorize this information through an additional spatial perspective, while haptic feedback influences the learning curve of manual dexterity (Wang et al., 2015). VR haptic simulation could also be beneficial to train experienced operators before facing complex cases. As shown in this case report, the opportunity to upload and combine in the simulator dicom data and stl files of the patient from CT scans and intraoral scanning, respectively, could allow the creation of the virtual specific patient case. Thus, the operator can preoperatively train on a challenging clinical case until reaching the desired level of accuracy and confidence. For this reason, VR haptic simulation is gaining increasing interest in continuing education programs or certification procedures. However, the primary setting of a VR haptic simulation room is definitely affected by relevant costs, and accessibility is still questionable for global adoption.

Although there are evident advantages to preclinical training with VR simulators, modern haptic devices only provide realistic tactile force feedback related to hard tissue management (enamel, dentin, caries, bone, root apex), while soft tissue procedures (flap incision and elevation, suturing) are still too sophisticated to be reproduced in a VR environment with reliable tactile feedback. This initial VR haptic exercise in modern EMS has the limitation of providing an interactive tactile experience only in ostectomy, apicoectomy and ultrasonic retro preparation. Improvements in haptic technology are needed for a reliable simulation of soft tissue management.

Further limitations emerged during the creation of the VR haptic simulation presented here. To generate a haptic object, a voxelization algorithm was utilized to transform surfaces into voxel‐made 3D objects, along with a smoothing algorithm to enhance realistic tactile feedback. A compromise had to be found between the minimum voxel size and the performance levels of the simulation. The smaller the voxels, the closer the generated haptic object would be to reality; however, this significantly affects the computational time, thus impacting the VR haptic stability and the quality of feedback. The same considerations were applied in setting the minimal buccal bone thickness (1 mm) to provide realistic tactile feedback during ostectomy in the VR scenario.

The current digital workflow for creating a new VR haptic scenario through the editor interface is still complex and time‐consuming and requires the professional intervention of an expert graphic software engineer. The process starting from zero implies: the creation of the 3D avatar of the patient, including mouth animations, etc. (2–4 months); the creation of the virtual haptic object from the real patient case (2–3 weeks depending on the quality and complexity of the sources and available data, and how much the process can be automated); fine tuning of the haptic feedback, of the power of tools and of the densities of different tissues after segmentation (2–3 weeks). Of course, adding a new case at the actual technological stage would probably take only a couple of weeks since there is no need to create a new patient 3D avatar and fine tuning of the main parameters is already set.

The ideal future goal is to implement a virtual simulator with automation software tools that allow for the immediate virtualization and simulation of an individual patient case by simply uploading the cone beam‐CT and intraoral scanner data. Other long‐term ongoing tasks are: the soft tissue management haptic feedback (flap incision, elevation and retraction, compression and suture); the improvement of the VR experience with a more realistic magnification effect and blood simulation; a better interaction through real‐time feedback to the student: in case of accidental damage to a site that is not supposed to be touched, a popup displaying a warning could appear. It would also be beneficial to upgrade the simulation of the retrofilling procedure, which usually requires significant manual dexterity to perform.

Furthermore, integrating artificial intelligence with the vast amount of data arising from the virtual haptic experience could allow for operator profiling, leading to more effective personalized learning and skill improvement. In addition, to ensure patient safety, it would be optimal to introduce Enstrustable professional activities (EPA) assessment to establish when trainees are ready for independent practice and less supervision. Using EPAs, the clinical activity context can be adjusted according to the trainee's progress by increasing patient complexity and environmental factors, at their individual rate of development (Kelly et al., 2022).

In conclusion, this case report demonstrates the implementation of VR haptic digital teaching into the endodontic curriculum as an active learning and training experience. Although further studies on similar procedures are needed to provide more comprehensive data, this report conceptualizes the potential to harness VR haptic technology in Endodontics.

## AUTHOR CONTRIBUTIONS

Damiano Pasqualini: conceptualization, VR haptic simulation setting, supervision of clinical procedure, writing (original draft preparation and editing); Giorgia Carpegna: conceptualization, writing (reviewing); Mario Alovisi: supervision; Elio Berutti: conceptualization, supervision; Sami Chogle: conceptualization, writing (reviewing).

## CONFLICT OF INTEREST STATEMENT

The authors declare that they have no competing interests regarding this article.

## ETHICS STATEMENT

The authors deny any conflicts of interest related to this study.

## Supporting information


Data S1:

Video S2:

## Data Availability

Data that support the findings of this study are available upon request from the authors.
