# Supplementary material for: Virtual reality and haptic simulation in modern microsurgical endodontics: Case report and proof of concept
Source: Int Endod J. 2025 Apr 19;59(6):1286–96. doi: 10.1111/iej.14239 (PMC13158539; doi:10.1111/iej.14239)
Supplement: Supplementary file 1 — Data S1: [file IEJ-59-1286-s001.pdf]

POSTURE

Aspect of the exercise	Grading options	Reasoning	Action Point
**My posture during the exercise**	Was optimal	Because the patient and I were in an ergonomic position	N/A
	Requires improvement	Because the patient position was incorrect	Position the patient appropriately
		Because I was not sitting in an ergonomic position	Establish a comfortable and ergonomic position before starting the exercise

OSTECTOMY			
**Aspect of the exercise**	**Grading options**	**Reasoning**	**Action Point**
**My ability to follow the prescribed outline of the ostectomy**	Was optimal	Because I was sitting in a good position, established a stable finger rest and maintained a stable fingers rest and maintained good handpiece control	N/A
	Requires improvement	Because my vision was impeded due to the position I selected	Establish a good working position to allow for complete visualization of working area
		Because I didn't establish a stable finger rest	Find and maintain a stable finger rest before starting the exercise
		Because I didn't maintain control of the handpiece	Ensure your grip of the handpiece is appropriate and your body in a comfortable and sustainable position
**Did I make any errors during the ostectomy?**	I made no errors	Because I established a stable finger rest and used the correct bur to the desired depth	N/A
	Yes, I made some errors	Because I seated the bur too far	Understand the dimension of the bur used and fully seat the bur to desired depth
		Because I did not seat the bur fully	Understand the dimensions of the bur used and fully seat the bur to desired depth
		Because I didn't establish a stable finger rest	Find and maintain a stable finger rest before starting the exercise

ROOT END RESECTION			
**Aspect of the exercise**	**Grading options**	**Reasoning**	**Action Point**
**How do you evaluate the extension and quality of the root end resection?**	Was optimal	Because I had the bus fully seated at the desired depth, angled the bur so it was parallel to the long axis of the tooth	N/A
	Requires improvement	Because I had more than one entry and one exit point for the bur	Ensure you have only one entry and one exit point for the bur
		Because I did not seat the bur fully	Understand the dimensions of the bur used and fully seat the bur to desired depth
		Because I did not follow the prescribed outline down the long axis of the toot.	Ensure your bur angulation is correct so that it will prepare the desired outline along the long axis of the tooth
**How do you evaluate the extension and quality of the root end bevel?**	Was optimal	Because I established a stable finger rest and used the correct bur to the desired depth	N/A
	Requires improvement	Because I didn't maintain control of the handpiece	Ensure your grip of the handpiece is appropriate and your body in a comfortable and sustainable position
		Because I didn't establish a stable finger rest	Find and maintain a stable finger rest before starting the exercise

RETRO CAVITY PREPARATION			
**Aspect of the exercise**	**Grading options**	**Reasoning**	**Action Point**
**How do you evaluate the extension and quality of the ultrasonic preparation?**	Was optimal	Because I had the ultrasonic tip parallel to the long axis of the tooth	N/A
	Requires improvement	Because I had more than one entry and one exit point for the ultrasonic tip	Ensure you have only one entry and one exit point for the bur
		Because I did not follow the prescribed outline down the long axis of the toot.	Ensure your tip angulation is correct so that it will prepare the desired outline along the long axis of the tooth
**My ability to follow the prescribed outline of the retro preparation**	Was optimal	Because I was sitting in a good position, established a stable finger rest and maintained a stable fingers rest and maintained good handpiece control	N/A
	Requires improvement	Because my vision was impeded due to the position I selected	Establish a good working position to allow for complete visualization of working area
		Because I didn't establish a stable finger rest	Find and maintain a stable finger rest before starting the exercise
		Because I didn't maintain control of the handpiece	Ensure your grip of the handpiece is appropriate and your body in a comfortable and sustainable position
